# Long-term psychiatric inpatients’ perspectives on weight gain, body satisfaction, diet and physical activity: a mixed methods study

**DOI:** 10.1186/s12888-018-1878-5

**Published:** 2018-09-18

**Authors:** Susanna Every-Palmer, Mark A. Huthwaite, Jane L. Elmslie, Eve Grant, Sarah E. Romans

**Affiliations:** 1 Department of Psychological Medicine, University of Otago Wellington, PO Box 7343, Wellington, 6242 New Zealand; 2 Capital and Coast District Health Board, Wellington, New Zealand; 3 University of Otago Christchurch, Christchurch, New Zealand

**Keywords:** Obesity, Serious mental illness, Physical health, Physical activity, Schizophrenia, Health promotion

## Abstract

**Background:**

Obesity is a significant problem for people with serious mental illness. We aimed to consider body size from the perspective of long-stay psychiatric inpatients, focussing on: weight gain and its causes and impacts; diet and physical activity; and the perceived ability to make meaningful change in these domains.

**Method:**

A mixed methods study with 51 long-term psychiatric forensic and rehabilitation inpatients using semi-structured interviews combined with biometric and demographic data.

**Results:**

94% of participants were overweight or obese (mean BMI 35.3, SD 8.1). They were concerned about their weight, with 75% of them attempting to lose weight. Qualitative responses indicated low personal effectiveness and self-stigmatisation. Participants viewed their weight gain as something ‘done to them’ through medication, hospitalisation and leave restrictions. A prevailing theme was that institutional constraints made it difficult to live a healthy life *(just the way the system is*). Many had an external locus of control, viewing weight loss as desirable but unachievable, inhibited by environmental factors and requiring a quantum of motivation they found hard to muster. Despite this, participants were thoughtful and interested, had sound ideas for weight loss, and wished to be engaged in a shared endeavour to achieve better health outcomes. Consulting people as experts on their experiences, preferences, and goals may help develop new solutions, remove unidentified barriers, and improve motivation.

**Conclusions:**

The importance of an individualised, multifactorial approach in weight loss programmes for this group was clear. Patient-led ideas and co-design should be key principles in programme and environmental design.

## Background

People with serious mental illnesses (SMI) such as schizophrenia, bipolar disorder and major depressive disorder, have poorer physical health and significantly reduced life expectancy compared with the general population [1]. Those with schizophrenia have a 2–3 fold increased standardised mortality ratio [1–4], a sobering fact aptly described as “the scandal of premature mortality” [5]. While the causes of this reduction in life span are multifactorial, there is little doubt that obesity and its complications (diabetes, heart disease, and hypertension) are significant contributors [6, 7].

However, amongst all the research on obesity and its correlates in SMI, very little traverses the experiences of those personally affected. A literature search we conducted in June 2017 revealed nearly 4000 articles containing the keywords ‘psychiatric disorder’ and ‘obesity’, but only a handful of qualitative studies containing personal accounts of those with SMI. The empirical evidence has incontrovertibly established a high prevalence of overweight and obesity *in* people with SMI [6–9], but the voices of people *with* SMI are lacking. The parlous physical health outcomes are well quantified, but what about effects of weight on self-esteem, confidence and stigma, and beliefs about causes, barriers and ideas for improved health and weight loss?

This apparent research gap is surprising, given the importance placed on patient-centred care and co-design in contemporary practice. Munk-Jorgensen and colleagues, for example, have noted the importance of involving patients in all stages of medical research, quoting the European Science Foundation [10].

The articles we identified covering mental health services users’ experiences of weight included a 2011 review of studies on the perception of patients and/or nurses of health promotions targeting physical activity and eating habits [11]. This review found that in general, both mental health patients and nurses held positive views about health promotion but patients felt the ability to improve their physical health was beyond their control. Several barriers to health improvement were identified, including the mental illness itself, medication side effects, lack of support, finances, stigma and, additionally from the nurses only, poor motivation and unwillingness to participate.

Blanner Kristiansen and colleagues examined patients’ and nurses’ perspectives on health promotion [12] as part of systematic interventions aimed at reducing health risks in people with schizophrenia living in the community. The views of staff and patients were aligned, with perceived causes for poor physical health categorised into three clusters: lifestyle, the mental disorder and organisational issues. Both groups wanted less fragmentation of the ‘treatment system’ with physical health issues incorporated into routine psychiatric care.

The other papers also used qualitative methods to study health attitudes of people with SMI. Blomqvist and colleagues identified being seen as a whole human being (rather than a psychiatric patient) by self and others as the central theme in achieving a healthy lifestyle [13]. Sub-categories of this theme were having a normal everyday structure (healthy daily activities, sleep, diet), life events and social support, which provide motivation for healthy habits. Another Swedish study examined the lived experiences of people with SMI making or contemplating life style changes [14]. They found that the most helpful interventions focussed on individualised strengthening of the person’s self-efficacy.

In an attempt to address the research gap, we have undertaken a mixed methods study exploring the experience of obesity and its correlates in a sample of long-term inpatients in New Zealand. Basic demographic and clinical data have been published already from an earlier project which quantified calorie intake, energy expenditure and sleep in the same cohort [15]. There we reported high calorie consumption with frequent non-nutritional eating (junk food), excessive somnolence (median total sleep time > 9 h) and low levels of physical activity in this sample. This study seeks to understand the subjective perspective of our participants about their weight, diet, exercise regimes and self-esteem.

## Methods

We used a mixed methodology approach: cross sectional data collection to quantify obesity and thematic analysis to explore the related experiences of our participants.

Eligibility criteria were English-speaking adult patients (> 18) residing in regional New Zealand forensic and rehabilitation services who were competent to provide informed consent. While all the patients included in this study had serious mental illness, this was not specifically an inclusion criteria, rather a consequence of the sampling frame (having a SMI is essentially a prerequisite for long term psychiatric inpatient rehabilitation in New Zealand).

The treating team determined capacity, which was reconfirmed during the informed consent process. The research setting comprised five different units across two geographical sites. Three units were medium secure, one minimum secure and one was an unlocked unit. Residents had access to a general practitioner, gymnasium equipment, circuit training, a personal trainer, and occupational therapy programmes. Some but not all participants could use a swimming pool, yoga classes, walking and healthy lifestyle groups, cooking and shopping training. Meals were supplied by the hospital kitchen, but many residents purchased and consumed supplementary food.

### Data sources

Sociodemographic and clinical information was extracted from clinical records and from the participants and included diagnosis, age, gender, ethnicity, date of admission, admission BMI, current BMI, smoking status, medication, age of first treatment onset and whether people were under mental health compulsory treatment orders.

Participants were interviewed about their weight, diet, activity levels, their ability to change these things and the impact on their self-esteem, using structured and semi-structured interview schedules.

The semi-structured interview schedule had five main probes and canvassed views about weight, diet and exercise. Participants were asked to describe:

- their feelings about their weight, how their weight had changed and what factors they saw as responsible for any weight change;
- any efforts they were making to change their weight;
- what changes, if any, they would make to their diet to maintain a healthy weight;
- whether they used the exercise resources available and what their thoughts and feelings about these resources were;
- how their physical activity differed in hospital compared to when they were living in the community.

The structured interview schedule collected demographic information and included the following three scale-based survey items:

- likert-scale item relating to body dissatisfaction;
- a three point scale on food volume (too much, right amount, not enough);
- dichotomous questions on non-hungry eating (eating driven by emotional factors or food availability despite being satiated).

The interviews were structured in ways that previous experience suggested participants would be most comfortable; one-on-one interviews in a private space on the hospital campus with a researcher known by the participants*.* Each interview took approximately 40 min. The interviewer transcribed responses during the interviews; manual transcription was more acceptable to the participants than audio recording.

### Analysis

Data from the quantitative items were entered into Excel 2013 and SPSS version-21 (SPSS Inc. Chicago, Illinois, USA). Descriptive statistics used appropriate tests for the data type.

Analysis was conducted in accordance with Braun and Clarke’s thematic approach [16]. Initially three researchers (SR, JE and MH) independently familiarized themselves with the interview transcripts. Data was then indexed in terms of similarity and contrast of content and initial codes were generated. These codes were then examined and discussed by the research team, moving back and forth between the data and the thematic coding, and were further condensed into themes and subthemes through this iterative process.

All recorded data was de-identified to preserve confidentiality.

### Ethics approval

The New Zealand Health and Disability Ethics Committee (reference 13/CEN/153) approved this study.

## Results

Fifty-one participants were recruited from a group of 87 eligible persons, a 59% response rate. They all had SMI, with onset of treatment (a proxy for illness onset) between 15 and 29 years previously. All participants had diagnoses of psychotic disorders, most commonly schizophrenia (78%), with diagnosis codes derived from data submitted to the national mental health database (PRIMHD) according to ICD 10 criteria. Most participants had been in hospital for over a year, and all but one were detained under a compulsory treatment order. Participant characteristics are summarised in Table 1.

**Table 1:** Participant characteristics

Sociodemographic factors		
Age	Mean = 38 years (SD 10.4, range 19–68)	
Gender		
Male	40 (78%)	
Female	11 (22%)	
Ethnicity		
New Zealand Pakeha	15 (29%)	
Māori	33 (65%)	
Pacific Island	13 (26%)	
Other	3 (6%)	
Relationship status		
Single	40 (78%)	
Married or de facto relationship	4 (8%)	
Separated or divorced	6 (12%)	
Widowed	1 (2%)	
Education		
No school leaving certificate	33 (65%)	
School certificate	14 (27%)	
Further higher qualifications	4 (8%)	
Illness-related factors		
Diagnosis (per ICD criteria)		
Schizophrenia	40 (78%)	
Schizoaffective disorder	6 (12%)	
Bipolar disorder (with psychotic features)	2 (4%)	
Other (unspecified psychosis etc.)	3 (6%)	
Legal status		
Voluntary inpatient order	1 (2%)	
Compulsory treatment order	50 (98%)	
Most common medications		
Clozapine	25 (49%)	
Non-clozapine antipsychotic	FGA = 4 (8%), SGA = 17 (33%), FGA + SGA =4 (8%)	
Mood stabiliser	15 (29%)	
Metformin	23 (45%)	
Statin	15 (30%)	
Mean age at onset of first treatment	21.9 years (SD = 6.7)	
Mean duration of treatment	15.9 years (SD = 9.4)	
Mean length of admission	19.7 months (SD = 20.3)	
Weight		
Mean BMI (kg/m^2^)	Our participants	New Zealand population [35]
All	35.3 (SD 8.1)	28.2 (SD 0.1)
Male	34.2 (SD 7.1)	28.1 (SD 0.2)
Female	39.4 (SD 11.0)	28.3 (SD 0.2)
World Health Organisation weight classification grading	Our participants	New Zealand population [35]
Normal weight range (BMI < 25)	3 (6%)	33.3%
Overweight (BMI 25–29.9)	10 (20%)	35.2%
Obesity Class I (BMI 30–34.9)	10 (20%)	18.9%
Obesity Class II (BMI 35–35.9)	14 (28%)	7.6%
Obesity Class III (BMI > 40)	13 (26%)	5.1%
Change in weight since admission		
Weight gain (> 5 kg)	24 (47.2%)	
Minimal change	14 (27.8%)	
Weight loss (> 5 kg)	13 (25%)	

### Weight

Obesity was the norm in our sample, with participants markedly heavier than the general population. Three quarters were obese with a mean BMI of 35.3 (SD 8.1), males 34.2 (SD 7.1), females 39.4 (SD 11.0, *p* = 0.07). Only three were of normal weight and none were underweight. The BMIs of our participants differed significantly from the national average. New Zealand has an obesity rate of 31.6% (χ^2^ = 17.084, df 1 *p* < 0.0001) and a mean BMI of 28.2 kg/m^2^ (observed difference 7.1 kg/m^2^, 95% CI 4.9–9.3, *p* < 0.00001).

Most participants reported gaining weight since starting antipsychotic medication (mean reported weight gain 33.4 kg).

### Personal views about weight

For causes of weight gain, 30 (60%) identified medication, 18 (36%) nominated food volume, 10 (20%) food type, 10 (20%) lack of exercise one illness and one smoking. One mentioned his use of synthetic cannabinoids as appetite stimulating (‘*the munchies’)* leading to over-eating.

Some reported being surprised by the extent of their weight gain, feeling it did not make sense in the context of their personal circumstances:

*‘Maybe a little bit of it is food but I don't eat that much, I hardly eat because I don't like the food here.’*

Only 21 (of 51) offered more than one cause of high body weight and none offered more than two (10: both food and medication, eight: food and lack of exercise, three: lack of exercise and medication). This suggests participants may hold a single cause (either-or) way of thinking about this issue.

While physical reasons predominated in response to survey questions about causes of weight gain, participants also had significant beliefs about psychological and environmental reasons underpinning their weight, which were strong themes in the semi-structured interviews.

### Weight and self-perception

Most people were unhappy with their weight. Three quarters (38/51) reported trying to lose weight. More than half worried about their body shape or appearance ‘some to all of the time’ (see Table 2). Over 60% reported being self-conscious and worried about their body shape when they were with other people. A strong theme of shame and self-condemnation emerged for some, in which their weight triggered negative feelings about themselves. Comments included: *‘being lazy’; ‘being a pig and eating too much’* and *‘being undisciplined’.*

**Table 2:** Body satisfaction

Item	Positive response (some-all of the time) *n* (%), *n* = 42	Male *n* (%) *n* = 31	Female *n* (%) *n* = 11	Significant difference between male and female
*Are you worried about your body shape or your appearance?*	26 (62)	20 (65)	6 (55)	ns
*Do you think your body shape is worse than other people’s: do you compare yourself negatively to others’ body shape?*	21 (50)	15 (48)	6 (55)	ns
*Do you become self-conscious and worried about your body shape when around other people?*	26 (62)	19 (61)	7 (64)	ns

Over-eating in response to emotional factors such as sadness or loneliness also emerged.

*‘Depression—eating due to emotions as girls do.’*

*‘Food, just wanting food—not being able to see my family.’*

This theme was reinforced by the survey questions on non-hungry eating (Table 3) in which almost half the participants (43%) endorsed eating in response to negative emotions. Females were significantly more likely to eat when emotionally distressed.

**Table 3:** Eating when not hungry

Questions	Yes (%)	Male Yes (%)	Female Yes (%)	Significant difference between male and female
*Do you have the urge to eat even when you’re not hungry?*	19 (45)	14 (45)	5 (45)	ns
*Do you find it hard to stop eating even when you are full?*	16 (38)	11 (35)	5 (45)	ns
*Do you realise that you have eaten more than you intended to?*	32 (76)	24 (77)	8 (73)	ns
*Do you eat more if there is food around?*	28 (67)	19 (61)	9 (82)	ns
*Do you eat when you are emotionally stressed, upset, worried, tense or bored?*	18 (43)	10 (32)	8 (72)	χ^2^ = 5.4, df 1 *p* = 0.03

Not everyone was unhappy with their body size. A minority reported being comfortable with their current weight ‘*I’m happy how I am’, ‘I want to be about 120kg but I want it to be muscle–I’m exercising in my room.’*

One male talked about the perceived advantages of being overweight in terms of personal protection and power, describing his weight gain as a deliberate strategy related to *‘needing to protect myself, for advantage over others and to feel good. I wanted to be bigger than my brother.’*

### Weight loss strategies

For those trying to lose weight, 32 (63%) identified exercise and 16 (31%) dietary modifications as the means to achieve their goal, with twelve noting both strategies.

### Diet

Most people (35/51) made suggestions for improvements in the hospital menu and in their own dietary choices. Thirty-one commented about some aspect of their food intake; these comments ranged from portion sizes to food type.

Suggestions included: ‘*eat more fruit’, ‘cut out bread’, ‘tick the medium size meal instead of large size’*, *and ‘cut down on my coke intake’.* Some recommended modifications to the hospital menus (‘*maybe some better recipes – it’s on a two-week rotation; getting more variety would be good’, ‘more vegetarian meals and [I wish] that the ones they serve were nicer’*).

Twenty-seven participants (56%) thought they were offered the right amount of food, 13 (26%) thought ‘not enough’ and nine (12%) ‘too much food’. There was no statistical association between BMI and perception of food adequacy. Gender was not significant although none of the 10 women considered that they were being offered too much food.

### Physical activity

Most people were participating in some activities provided on the campus (e.g. playing table tennis, volleyball, swimming, joining the walking group, using the personal trainer). Seven specified ‘using the gym’. Ten (21%) said that they used the courtyard daily. Nine indicated that no courtyard was available (one unit’s courtyard was being renovated) and three needed staff to be available to access the space.

Eighteen participants, 14 men (45%) and four women (36%) said they used the personal trainer regularly. Three indicated that they did not like to use weights and so did not work with the trainer. Some gave practical reasons for not using the personal trainer (three needed a referral, one had a conflicting appointment with the social worker at that time). Four indicated that the personal trainer was not available in their area.

There were some intriguing responses, which suggest underlying difficulties with working with the trainer, such as low motivation or frank, active psychotic symptomatology. Comments included: *‘I don’t want to see her’, ‘sick of that sort of thing’, ‘decided to stop going because of the voices’.* Some idiosyncratic plans for weight management included secrecy around exercise: *‘I am on my own plan for my work out but it’s a secret’* and *‘[I’m] running in the shower’.*

### Activity levels in hospital compared to their community living

Forty participants (78%) said that they had been more active in the community than as inpatients and only nine indicated more activity in hospital. A number of the forensic patients spontaneously volunteered that they had been more active in prison. While this may be subject to recall bias, many provided credible information to illustrate their response with the following examples:

*‘I only get to go out a certain number of times/hours compared to the community when you can go out any time.’*

*‘Yeah - when in the community, I would usually walk around and up town to my mate’s place everyday.’*

*‘I’m currently doing way less than when in community and in prison. There is nowhere to go to get out.’*

*‘I used to run for an hour every day in prison. I’ve just started getting back into this.’*

*‘You can't do what you want to do in here, but outside you can.’*

*‘We do nothing in comparison to the outside where you're working and motivated.’*

### Barriers

Despite the options available for exercise, participants perceived significant barriers to weight management. People felt the highly regulated environment, with restrictions on personal autonomy, which one described as *‘just the way the system is’,* contributed to both their weight gain and their inability to shift this weight. Some comments suggested an external locus of control and feelings of disempowerment. Other responses addressed systemic issues and including the restriction of movement, commenting on ‘*unfair treatment’*. Frequent reference was made to leave restrictions as a barrier to an active life.

Some participants specifically mentioned *‘low motivation’*. Many described low self-efficacy to eat well or persevere with physical activity, with at least some showing self-awareness of these characteristics. *‘Don’t go [to the gym], cos I can’t be stuffed’,* and *‘I used to [meet with the personal trainer] but don’t go anymore as I don’t feel like it.’*

Participants identified obstacles that, in their view, made exercising difficult:

*‘I sleep too much as the medication is too strong.’*

*‘We don't have a ball.’*

*‘I used to have leave to the courtyard but it got stopped ‘cos I was getting high out there.’*

*‘I have access for walks around [the courtyard] in the morning and when the weather is ok, we play volleyball. However, I have got holes in my shoes.’*

A number of comments showed the crucial importance of the socially interactive aspect of physical activity.

*‘I don't, ‘cos no one else does.’*

*‘I’d rather stay inside and see all the drama and action.’*

*‘It’s boring out there on my own.’*

## Discussion

This study focused on a particularly disadvantaged population: people with a mental illness serious enough that it necessitated a long period of inpatient psychiatric rehabilitation, the vast majority of whom were detained under compulsory mental health act legislation. This is a group whose opinions are seldom canvassed. In the review from Verhaeghe’s group, only one (*n* = 12) of 14 studies of health attitudes in people with mental illness and their nurses had a specific inpatient focus [11]. The reviewed studies used qualitative methods, so our inpatient study with our combined quantitative and qualitative methods adds substantively to the existing literature.

### Participants were overweight and they cared about this

Obesity was a significant problem for our participants. Being overweight was not a selection criterion, but an overwhelming majority (94%) were overweight or obese. Participants were self-conscious and worried about their weight. Some described self-blame and self-disgust using derogatory epithets such as *pig* or *lazy* to described themselves. This group clearly carried society’s high stigma about obesity [17], a double jeopardy when combined with the stigma of SMI. This seemed to lead to personal passivity and nihilism. These results differed from those of Minsky and colleagues who found that people with SMI underestimate their obesity [18]. They do align with the findings of Blanner Kristiansen’s group that overweight was a paramount physical health problem for their participants [12].

### Overt and covert reasons for overweight

Thematic analysis of responses revealed participants implicated physical, psychological and social factors within the hospital as promoting and maintaining their weight gain. The physical factors were overt, explicitly identified by participants as causal factors for obesity, and included medication, diet, institutional restrictions and the sedentary lifestyle. Most participants viewed the combination of weight-promoting medication, inactive lifestyles, (most apparent in those patients with a higher security status and no leave), and diet as having potent effects on weight. They considered the inpatient environment inhibited incidental activity, such as active paid jobs, or walking in the community as a means of getting somewhere.

The psychological and social factors were more implicit and included loneliness, isolation, pleasure seeking and social isolation effects. Participants talked about using food to manage their feelings and as a substitute for other comforts. Narratives highlighted their lack of normal hedonic activities: warm, affectional bonds with close family members, including but not limited to sexual connectedness and recreation (socialising with friends, community activities, pursuit of hobbies and holidays and travel). The reduced access to previously used rewards such as alcohol may also be relevant. In a psychologically arid inpatient environment, food may have become overly important, becoming a substitute for other gratification sources.

A strong theme for the forensic participants was weight gain and reduced physical activity after moving from prison to hospital. Being fit and strong has high salience in custodial settings but less so in psychiatric hospitals. While medication and illness factors cannot be disentangled, social networks are known to influence weight status. The Framingham Heart study demonstrated that when a close social contact becomes obese, an individual’s likelihood of similar weight gain increases by 171% [19]. In our setting, being surrounded by overweight people might change a person’s tolerance for obesity and lead to imitation of weight promoting behaviours. Participants themselves clearly identified peers’ behaviours as impacting on their own exercise regimes *(I don’t, ‘cos no one else does).* Eating habits may have been similarly influenced.

Overall, participants found the hospital’s environmental constraints made living a healthy life difficult, and thought that changing this was beyond their control (*just the way the system is)*. This may be a consequence of the well-recognised concept of institutionalisation, defined as “the impoverishment of feelings, thoughts, initiative and social activity” [20] which arises as a possible consequence of long term residence in a confined and controlled environment [21]. Social isolation and dependence on staff may result in the loss in belief of one’s ability to act autonomously and in disempowerment. This is often compounded by illness-related factors. Maintaining the commitment and drive necessary for long-term weight loss is challenging for anyone, but even more so for those with SMI. Motivational deficit is a core negative symptom of schizophrenia [22]. Low motivation reduces the ability to predict future rewards, as well as the ability to appreciate or enjoy pleasurable experiences in the present (anhedonia) [23]. This may lead to an anhedonic drive to eat, with low motivation to change eating behaviours or physical activity.

Sørensen’s research team [24] found people with SMI were more physically active if they possessed intrinsic motivation, held a cognitive self-schema of themselves as a physically active person, and found the activity to be enjoyable. Ussher and colleagues interviewed 120 people with SMI about physical activity preferences and barriers, and found a high level of interest in exercise but low ‘self efficacy’ (confidence in their ability) to exercise when sad or stressed [25]. Barriers included lack of support from family and friends, fatigue, illness, and bad weather. Medication sedation, weight gain, fear of unsafe conditions, fear of discrimination, lack of understanding, social and cultural factors (e.g., social isolation), and the physical environment and policy have also been identified as barriers [26, 27]. Our results echo those findings.

### Recommendations and future research

Holistic approaches, with individualised strengthening of self-efficacy have been recommended [13, 14]. Sørensen’s construct of internal cognitive schemas, anchored but potentially mutable, seems useful [24]. If core beliefs of being lazy, greedy or weak could be reshaped through psychological interventions to become schemas of being active and healthy people, motivation could potentially be enhanced.

Further research could investigate “nudges” using behavioural economic approaches, focussing on interventions that make healthier choices seem easier and more rewarding. For example, present bias (overvaluing immediate costs and benefits and undervaluing future costs and benefits [28]) could be utilized with programmes that offer small, frequent (and therefore immediate) rewards for beneficial behaviours [29]. Such programmes targeted at weight loss, medication adherence and smoking cessation work in general community samples [30–32]. Newer technology such as fitness trackers warrants further investigation. For someone wanting to lose weight but struggling with motivation, the instant gratification of a daily steps tracker is more rewarding than deferred and subtle changes on the scales. More emphasis on the short and medium term benefits of exercise on mental health [33] may also be helpful.

Although staff at our research sites were cognisant and concerned about the health problems of residents, this does not suffice. While the services offered resources, many patients felt they were passive participants rather than active collaborators in choosing and using these resources. Targeting the obesogenic environment through lifestyle interventions for people with SMI has been shown to reduce waist circumference and metabolic risk in the short term, but sustaining these improvements is challenging and requires ongoing commitment [34]. Services need to think about how the environment can support healthy diets and physical activity—both deliberate (active healthy choices) and incidental (e.g. physical activity as a part of normal activities of daily living). Environmental (social and psychological) aspects are important. An inpatient environment with appealing, well-equipped accessible exercise and outdoor spaces designed by and for the users are necessary. Physical activity should be enjoyable and reflect the individual’s preferences and include social features. Exercising with family, peers or support workers who embody healthy behaviour can be a powerful motivator and makes activities more fun and sustainable*.* Therapeutic attention to the type, amount, and variety of food provided is also important, as is the emotional and psychological drivers of overeating.

The commonly held, oversimplified attribution of participants that weight gain had been ‘done to’ them by antipsychotic medication may lead to the view weight gain is inevitable and cannot be reversed without stopping treatment. This is potentially amenable with psychoeducation. Understanding that antipsychotic medication may promote appetite, but it is actually increased food consumption that leads to weight gain, allows for greater self-determination.

As a group, our participants were thoughtful and concerned about their health and weight, and when asked, were able to articulate clear, well-grounded ideas about their predicament, supporting the work of Blanner Kristiansen and her colleagues [12]. Participants had some very specific diet recommendations, such as more vegetarian options and greater variety than a static two week rotating menu. Our dietitian researcher (JE) liked the suggestion that large portion sizes not be routinely offered but only available on specific request.

### Limitations

As we drew our participants from only two hospital sites, findings may not be more widely generalizable. Similarly, the primary diagnosis for the majority of participants was schizophrenia; people with other diagnoses may have different experiences.

The study used a cross-sectional design, so it is not clear whether self-image and self-efficacy around health and well-being changes during extended inpatient stays. This should be studied specifically and could become an outcome metric for inpatient care.

Data saturation was not sought as all the interviews were conducted before the data analysis. Some emergent themes (such as the advantages of high body weight, for example as protection against victimisation from others) warrant further study. Some participants had lost some weight over their most recent admission, however in interviews these participants focussed more on their total weight gain since entering psychiatric services, rather than recent success in losing some of this gained weight. Re-interviewing this subgroup as to how they had lost weight and whether they had sustained this would have been of value.

## Conclusions

While we considered weight from the perspective of an inpatient group, many themes are more broadly applicable to mental health service users. Our participants were overweight, and cared about this, with 75% of them reporting attempts to lose weight. Self-blame and disgust were evident in some, alongside a reduced sense of control and personal efficacy. Participants attributed weight gain to a combination of physical, psychological and social factors. A prevailing theme was that the institutional constraints made it difficult to live a healthy life, and that changing this was beyond their control.

Overall, given the interest and range of imaginative ideas about strategies for weight loss, participants showed themselves worthy and engaged collaborators in the quest for better health outcomes. Consulting people as experts on their experiences, preferences, and goals may help develop new solutions, remove unidentified barriers, and improve motivation.

Patient led ideas and co-design should be key principles in programme and environmental design.

## Abbreviations

BMI: Body mass index
Df: Degrees of freedom
FGA: First generation antispychotic
Kg: Kilogram
*n*: Number
ns: Not significant
SD: Standard deviation
SGA: Second generation antipsychotic
SMI: Serious mental illness
